# Quantitative sensory testing for assessment of somatosensory function in children and adolescents: a scoping review

**DOI:** 10.1097/PR9.0000000000001151

**Published:** 2024-04-03

**Authors:** Perri R. Tutelman, Nicole E. MacKenzie, Christine T. Chambers, Siobhan Coffman, Laura Cornelissen, Brittany Cormier, Kristen S. Higgins, Jackie Phinney, Markus Blankenburg, Suellen Walker

**Affiliations:** a Department of Psychology and Neuroscience, Dalhousie University, Halifax, Canada; b Centre for Pediatric Pain Research, IWK Health, Halifax, Canada; c Department of Pediatrics, Dalhousie University, Halifax, Canada; d Department of Anesthesiology, Critical Care & Pain Medicine, Boston Children's Hospital, Boston, USA; e Department of Anaesthesia, Harvard Medical School, Boston, USA. Cornelissen is now with the Alzheimer's Disease and Brain Health, Eisai Inc, Nutley, NJ, USA; f Dalhousie Medicine New Brunswick, Dalhousie University, Saint John, Canada; g Pediatric Neurology, Psychosomatics and Pain Therapy, Children's Pain Center Baden-Württemberg, Klinikum Stuttgart, Germany; h Department of Paediatric Anaesthesia, Great Ormond St Hospital NHS Foundation Trust, London, United Kingdom

**Keywords:** Quantitative sensory testing, Pediatric pain, Somatosensory functioning, Scoping review

## Abstract

Quantitative sensory testing (QST) refers to a group of noninvasive psychophysical tests that examine responses to a range of calibrated mechanical and thermal stimuli. Quantitative sensory testing has been used extensively in adult pain research and has more recently been applied to pediatric pain research. The aims of this scoping review were to map the current state of the field, to identify gaps in the literature, and to inform directions for future research. Comprehensive searches were run in 5 databases. Titles, abstracts, and full texts were screened by 2 reviewers. Data related to the study aims were extracted and analyzed descriptively. A total of 16,894 unique studies were identified, of which 505 were screened for eligibility. After a full-text review, 301 studies were retained for analysis. Date of publication ranged from 1966 to 2023. However, the majority of studies (61%) were published within the last decade. Studies included participants across the developmental trajectory (ie, early childhood to adolescence) and most often included a combination of school-age children and adolescents (49%). Approximately 23% of studies were conducted in healthy samples. Most studies (71%) used only one QST modality. Only 14% of studies reported using a standardized QST protocol. Quantitative sensory testing in pediatric populations is an emerging and rapidly growing area of pain research. Future work is needed using comprehensive, standardized QST protocols to harness the full potential that this procedure can offer to our understanding of pediatric pain.

## Abstract 2

Supplemental Digital Content is Available in the Text.

The use of quantitative sensory testing (QST) in pediatric pain research has grown substantially. Future research using comprehensive, standardized QST protocols is needed.

## 1. Introduction

Quantitative sensory testing (QST) refers to a group of noninvasive psychophysical tests that examine responses to a range of calibrated mechanical and thermal stimuli. Quantitative sensory testing methods emerged from efforts to improve neuropathic pain assessment through sensory profiling to phenotype pain features, understand neurophysiologic function, and advance mechanism-based pain care.^5,21^ Typically, QST involves applying a calibrated stimulus to the skin, and recording the individual's perception. Stimulus modalities are generally mechanical (ie, monofilaments, pinprick, vibration) or thermal (ie, computer-controlled thermodes) and outcome measures include stimulus detection (present/absent) and stimulus intensity (self-reported rating).^6^ Patterns of hyposensitivity (elevated sensory thresholds) and hypersensitivity (lowered sensory thresholds) across modalities can occur because of injury or disease, including many types of neuropathic pain.^8,29^ Although QST has been most commonly used to assess patterns of sensory functioning (ie, sensory profiling), there has been increasing interest in more advanced, dynamic techniques such as those that allow for examination of inhibitory processes (eg, conditioned pain modulation [CPM])^23^ and central processes (eg, fMRI).^26^

Quantitative sensory testing allows for the evaluation and quantification of small- and large-fibre somatosensory function^34^ and has significantly advanced our understanding of the neurobiological mechanisms and associated psychosocial factors that underpin pain processing.^8,18,39^ Quantitative sensory testing has also been applied more broadly as a tool to assess the efficacy of novel pain interventions^1,21^ and as a technique to induce experimental pain in clinical research.^2,5^ Although QST has been used extensively in adult experimental pain research over the last several decades,^18^ it has more recently been applied to pain research in children.

Adult QST protocols are standardized and studied extensively to ensure valid and reliable results.^20,34^ Standardized protocols permit comparison of one individual to normative values and comparison to large-cohort patient populations.^6,34^ Efforts have been made to standardize QST protocols in children,^11,13,31^ but designing and implementing such studies comes with challenges. To date, literature on the use of QST in children has been summarized by one systematic review and meta-analysis, focusing on the specific application of QST in pediatric chronic pain and the relationship between QST measures and pain intensity and disability. Schoth et al.^35^ reviewed 60 studies and identified that children with chronic pain exhibited lower pressure pain detection thresholds compared with healthy controls. Correlations between pressure pain and pain intensity and functioning were found for children with headache and arthritis.^35^ There is growing recognition that somatosensory changes may underlie the experience of pain for children in a broad range of conditions, not just those traditionally considered to be chronic pain disorder,^22,38^ and QST can be applied to advance pediatric pain research beyond the evaluation of sensory profiles. However, there has not been a comprehensive synthesis on how QST has been used to assess somatosensory functioning more broadly across all pediatric populations. An overview of QST studies conducted with children is needed to summarize the current state of the field, to identify gaps in the literature, and to inform directions for future research.

To address the gaps in the literature, this scoping review had 3 primary objectives: (1) to map the extent and nature of empirical research using QST to assess somatosensory function in children and adolescents; (2) to identify empirical and methodological gaps in the literature (eg, populations studied, consistency and rigour of QST protocols, measurement of adverse events), and (3) to inform directions for future research using QST and to outline specific considerations and recommendations in reporting of QST protocols in children and adolescents.

## 2. Methods

This scoping review of the literature was conducted in accordance with the methodological framework outlined by Arksey and O'Malley and Levac,^3,28^ which involves a 6-step process. These steps involve: (1) identifying the research question, (2) identifying relevant literature, (3) study selection, (4) charting the data, (5) collating, summarizing, and reporting the articles, and (6) consulting and translating knowledge. Before initiating the review, a protocol was registered with the Open Science Framework.^37^

### 2.1. Search strategy

After the researchers (P.R.T., N.E.M.) conducted a preliminary search and identified key papers on this topic, a health sciences librarian (J.P.) created a robust database search strategy that included a variety of keywords and subject headings focusing on quantitative sensory testing (and its modalities), as well as pediatric populations. The search strategy was peer-reviewed by a second health sciences librarian using the criteria found in the PRESS checklist.^30^

The database searches were conducted in Embase (Elsevier), MEDLINE (Ovid), CINAHL (EBSCO), PsycINFO (EBSCO), and Web of Science Core Collection (Clarivate) in October 2019, April 2021, June 2022, and November 2023. The search strategies included filters for human subjects, when possible, and English-only results. For the search updates in April 2021, June 2022, and November 2023, publication date filters were applied within the database. All database results from the October 2019 search iteration were deduplicated in EndNote before being uploaded to Covidence systematic review software^17^ where further duplicates were removed. For the second, third, and fourth search iterations, all results were uploaded directly to Covidence for duplicate removal. As an institutional subscription to Web of Science (Clarivate) was not available for the third and fourth search iterations, that search strategy was run by a partnering library and exported results were merged with the rest of the review data. The full search strategies are available in the supplemental materials, http://links.lww.com/PR9/A227.

### 2.2. Study selection and data extraction

Titles and abstracts were screened by at least 2 independent reviewers (P.R.T., N.E.M., B.C., K.H., or S.C.) to identify studies that potentially met the inclusion criteria. The full texts of the included citations were assessed in detail against the inclusion criteria by at least 2 independent reviewers. Studies were included if they were (1) original research papers using mechanical and/or thermal stimuli to assess somatosensory function in children and adolescents (mean/median age of sample 18 years and under); (2) published in English in a peer-reviewed journal; and (3) reporting on sensory, protocol- or experience-related (eg, acceptability, feasibility) outcomes. Studies were excluded if they (1) evaluated cold tolerance as the sole sensory outcome (eg, studies exclusively using the cold pressor task as these have been thoroughly reviewed elsewhere^10^); (2) systematic or scoping reviews, case reports, commentaries, dissertations, conference abstracts, books, book chapters, and letters to the editor; (3) studies using invasive stimuli (eg, rectal or esophageal manometry); (4) studies on nonhuman subjects; (5) studies with outcome measures other than child self-report (eg, evoked potential, behavioral responsivity, withdrawal response); or (6) studies that did not include a clear or replicable testing protocol to evaluate somatosensory function.

**Figure 1.**
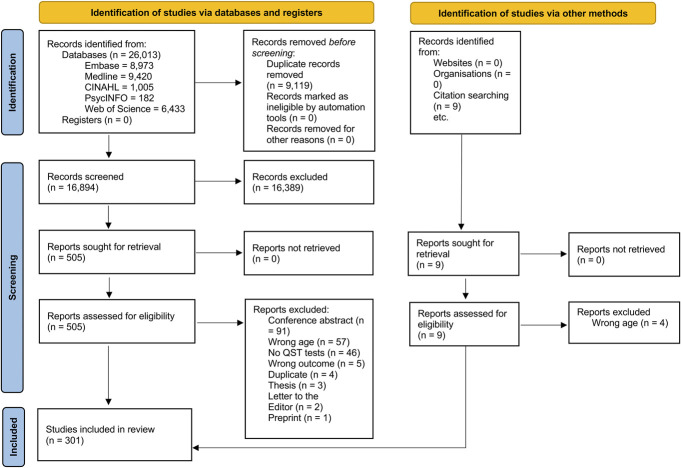
PRISMA flow diagram. PRISMA, Preferred Reporting Items for Systematic Reviews and Meta-Analyses.

Once screening of the database results was completed, the research team conducted backward searching of the reference lists of included studies. The 9 articles identified through backwards searching were incorporated into the Preferred Reporting Items for Systematic Reviews and Meta-Analyses (PRISMA) flow chart under “Identification of studies via other methods” (Fig. 1).

Data were extracted from the included articles by 2 independent reviewers (P.R.T., N.E.M., B.C., K.H., or S.C.) using a custom form that was developed and piloted by the research team. Data extracted from each study included study descriptors, study aims, study population, protocol information, and outcomes reported. Any disagreements that arose between reviewer pairs during study screening or data extraction were resolved by consensus.

Extracted data were synthesized with frequencies for the following variables: journal, year of publication, country of corresponding author, participant age and type, use of QST, sensory modalities, test location, sensory, feasibility, and/or acceptability outcomes reported, whether a standardized protocol was used and whether parents were present during the testing and summarized narratively. Results are reported in accordance with the Preferred Reporting Items for Systematic Reviews and Meta-analyses for Scoping Reviews (see supplementary materials for PRISMA checklist, http://links.lww.com/PR9/A227).^36^ Assessment of individual study bias is not typically conducted for scoping reviews^36^ and thus was not completed for the current study.

## 3. Results

### 3.1. Study selection

A total of 26,013 records were identified through multiple database searches. After removal of duplicates, 16,894 records were screened at the title or abstract level, of which 505 were retained for full-text review. Backward searching of full-text articles identified another 9 articles assessed for eligibility. After review of full-text articles and articles identified through backwards searching, 301 studies were retained for analysis. See Figure 1 for the PRISMA flowchart.

### 3.2. Study characteristics

**Figure 2.**
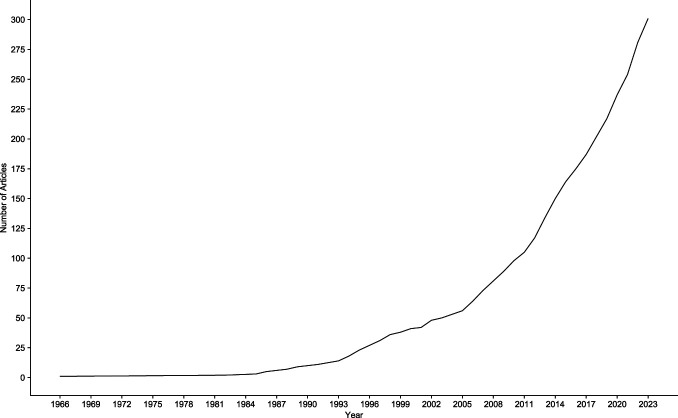
Number of publications over time.

The 301 articles that met the inclusion criteria were published in 161 distinct peer-reviewed journals. The 2 journals with the greatest number of publications were *PAIN* (n = 35, 11.63%) and the *European Journal of Pain* (n = 13, 4.32%). Year of publication ranged from 1966 to 2023; however, the majority of studies (n = 184, 61.13%) were published within the last decade (ie, since 2013; see Fig. 2). Corresponding authors were from 30 different countries. The majority (n = 241, 80.07%) were from the following 10 countries: the United States (n = 98, 32.56%), Canada (n = 28, 9.30%), Germany (n = 27, 8.97%), the Netherlands (n = 16, 5.32%), Denmark (n = 16, 5.32%), Brazil (n = 13, 4.32%), Spain (n = 13, 4.32%), Australia (n = 11, 3.65%), the United Kingdom (n = 10*,* 3.32%), and Israel (n = 9, 2.99%).

### 3.3. Participant characteristics

Children who participated in the included studies spanned the developmental trajectory, ranging from early childhood (eg, 2–5 years old) to adolescence (13–19+ years). A few studies included participants as young as 2 years of age for some QST tests.^4,33^ However, studies most often included a combination of school age (eg, 7–12 years) children and adolescents (ie, 7–19+ years; n = 146, 48.50%).

One-quarter of the studies (n = 67, 22.26%) were conducted with samples of generally healthy children (ie, no identified medical or developmental concerns) as the main sample of interest, whereas another quarter (n *=* 83, 27.57%) were conducted in children with various forms of chronic pain (eg, abdominal pain, headache). Approximately 17% of studies (n = 52) included participants with various chronic illnesses (eg, diabetes and sickle cell anemia). The remaining 32.89% of studies (n = 99) were conducted with other clinical populations including children with neurological disorders (eg, cerebral palsy, epilepsy, 6.31%), injuries (eg, nerve injury, burns, 3.99%), history of preterm birth or problems at birth (3.65%), orthopedic conditions (eg, scoliosis, 5.32%), neurodevelopmental disorders (eg, autism spectrum disorder, attention-deficit hyperactivity disorder, 4.32%), mental health conditions (eg, eating disorders, personality disorders, 2.99%), and postoperative populations (eg, hernia repair, cleft lip/palate surgery, 2.33%). A total of 10 studies (3.32%) included other distinct populations (eg, children with congenital deafness, offspring of parents with illness). Two studies were conducted with combined populations (ie, neurodevelopmental disorders and mental health conditions, 0.33%, and chronic pain and chronic illness, 0.33%). More than half of the studies (n = 176, 58.5%) included a control group for comparison. See Supplementary Materials for further detail on participant characteristics, http://links.lww.com/PR9/A226.

### 3.4. Protocol characteristics

The majority of studies (n = 259, 86.05%) used QST to evaluate sensory thresholds (ie, sensory profiling) in a given sample. Far fewer used a QST protocol for more advanced applications such as to examine inhibitory processes (ie, conditioned pain modulation; n = 35, 11.63%), development of normative values or evaluating test reliability (n = 34, 11.30%), to assess the efficacy of an intervention (n = 27, 8.97%), or to evaluate central mechanisms (eg, using fMRI, n *=* 13, 4.32%). In 3 studies (1.00%), the QST applications were something other than the categories above (eg, assessing the feasibility of a method, determining baseline pain). In 6 studies (1.99%), the purpose of using QST was not adequately described.

Of the 301 studies included, 28.90% (n = 87) reported using QST protocols that included both thermal and mechanical stimuli. The majority of studies (n = 159, 52.82%) used only mechanical stimuli, and approximately one-fifth of studies (n = 54, 17.94%) used only thermal stimuli. Across studies that used mechanical stimuli (n = 246), most examined participant responses to pressure (n = 153, 50.83%), followed by thin plastic filaments (eg, von Frey hairs, n = 71, 23.59%), vibration (n = 63, 20.93%), cutaneous punctate (eg, PinPricks, n = 42, 13.95%), light touch (eg, cotton wisp; n = 36, 11.96%), or other mechanical stimuli (n = 22, 7.31%). For studies that included thermal stimuli (n = 141), the majority used heat as the stimulus (n = 123, 87.23%), and approximately half examined participant responses to cold (n = 85, 60.28%).

Regarding test location, one-third of studies (n = 117, 38.87%) assessed participant responses to stimuli applied to the arm. Other common distinct test locations included the finger (n = 81*,* 26.91%), leg (n = 78, 25.91%), and thenar eminence (n = 60, 19.93%). Forty-five studies (14.95%) applied stimuli to a painful location on the body the child identified, and 13 studies (4.32%) applied stimuli to areas surrounding existing scars on the child's body. See Table 1 for further detail on test locations.

**Table 1:** Quantitative sensory testing test site grouped by major body areas.

Test site	*n* (%) (N = 301)
Head & neck	57 (18.94)
Face	43 (14.29)
Forehead	6 (1.99)
Neck	11 (3.65)
Upper extremity	227 (75.42)
Arm	117 (38.87)
Finger	81 (26.91)
Hand (thenar eminence)	60 (19.93)
Hand (dorsum)	30 (9.97)
Hand (not specified/other)	28 (9.30)
Thumb	3 (0.99)
Thorax	53 (17.61)
Back	49 (16.28)
Chest	5 (1.66)
Abdomen	10 (3.32)
Stomach	10 (3.32)
Lower extremity	115 (38.21)
Leg	78 (25.91)
Foot	51 (16.94)
Toes	28 (9.30)
Other	72 (23.59)
Identified painful location	45 (14.95)
Tender points	10 (3.32)
Scar	13 (4.32)
Other (eg, affected body site)	17 (5.65)
Location not reported	2 (0.66)

The total is greater than 301 as some studies tested multiple locations.

Across all studies, the most commonly reported sensory outcomes included sensory or pain thresholds (n = 260, 86.38%), followed by subjective pain ratings of QST tests (n = 109, 36.21%), sensory or pain tolerance (n = 45, 14.95%), perceptual sensitization (n *=* 37, 12.29%), inhibitory modulation (n = 32, 10.63%), allodynia (n = 21, 6.98%), thermal sensory limen (n = 11, 3.65%), paradoxical heat sensation (n = 10, 3.32%), or other (n = 3, 1.00%).

Approximately half of the included studies (n = 158, 52.49%) reported on feasibility or acceptability of the QST protocol. Feasibility outcomes included protocol completion rate (n = 101, 33.55%), protocol duration (n = 60, 19.93%), child's understanding or cooperation (n = 45, 14.95%), rates of equipment failure (n = 22, 7.31%), and adverse events (n = 17, 5.65%). Thirty-one studies (10.30%) reported on other aspects of feasibility (eg, procedure tolerance and experimenter error). Regarding acceptability, 8 studies (2.66%) reported on participant satisfaction. See Table 2 for further detail on feasibility and acceptability outcomes.

**Table 2:** Acceptability/feasibility outcomes.

Outcome	*n* (%) (N = 301)
Satisfaction	**8** (**2.66)**
No complaints	5 (62.50)
Described as challenging/interesting	2 (25.00)
Other (eg, positive experience, would return)	1 (12.50)
Adverse events	**17** (**5.65)**
None reported	10 (58.82)
Anxiety, fear, upset	5 (29.41)
Testing stopped because of fear of tissue contusion	1 (5.88)
Participant sustained a burn	1 (5.88)
Equipment failure	**22** (**7.31)**
Equipment failure or technical problems	20 (90.91)
Unavailability of equipment/software	1 (4.55)
Instrument slid off during testing	1 (4.55)
Understandability/cooperation	**45** (**14.95)**
Test duration	**60** (**19.93)**
30 min or less	20 (33.33)
Between 30 min and 1 h	19 (31.67)
Between 1–2 h	14 (23.33)
Between 2–3 h	4 (6.67)
Over 3 h	1 (1.67)
Reports individual task time only (vs total procedure)	2 (3.33)
Completion rate	**101** (**33.55)**
All participants completed the study	18 (17.82)
1 or more participants did not complete the study/were excluded	52 (51.49)
10 or more participants did not complete the study/were excluded	20 (19.80)
More than 50 participants did not complete the study/were excluded	4 (3.96)
Other	7 (6.93)
Other	**31** (**10.30)**
Procedure tolerance	11 (35.48)
Reported on feasibility	18 (58.06)
Experimental error	2 (6.45)
Not reported	**143** (**47.51)**

Bolded percentages represent the number of studies where the outcome was reported out of the total number of studies included in the review (ie, N = 301). Nonbolded percentages represent the number of studies where the finding was reported out of the total number of studies that reported that category of outcome.

A standardized QST protocol was cited in only 13.62% of included studies (n = 41), the most common of which was the German Research Network on Neuropathic Pain protocol (n = 25, 8.31%). Other commonly referenced protocols included those by Meier et al. (n = 8, 2.66%) and Van den Bosch et al. (n = 3, 1.00%). Furthermore, only 19.60% of studies indicated whether participants were allowed to watch the QST tests (n = 3, 1.00%) or were told to look away or wear a blindfold (n = 56, 18.60%). Over three-quarters of studies (n = 239, 79.40%) did not report whether parents were present or absent during the testing procedures.

## 4. Discussion

The overarching aim of this scoping review was to map the extent and nature of empirical research using QST to assess somatosensory function in children and adolescents. A total of 301 studies were identified. Although the earliest study was published in 1966, the vast majority have been published within the last decade. Findings suggest that the use of QST to assess somatosensory function in children is an emerging and rapidly growing area of research.

This review identified that approximately half of pediatric QST studies published to date have been conducted with samples of generally healthy children or with children with primary chronic pain. Indeed, the QST field emerged from efforts to characterize chronic neuropathic pain. However, there is mounting evidence supporting somatosensory changes across a range of common pediatric conditions not traditionally considered to be chronic pain disorders (eg, cancer, diabetes, and cerebral palsy), but that are still associated with significant levels of pain, pain-related disability, and risk for developing secondary chronic pain.^22,38^ Findings from this review suggest that, to date, QST has been applied narrowly in pediatric research with untapped potential in a range of childhood medical disorders and developmental disabilities. Cross-disciplinary efforts are needed to extend QST methods across pediatric populations. Furthermore, the majority of QST studies to date have been conducted with school-age children and adolescents. Fewer studies have included children younger than 7 years. Indeed, there are age-varying changes in sensory thresholds,^12^ which must be considered in analyses. The youngest age at which a child can feasibly engage in QST testing remains unclear. Although some protocols specify 6 years as the minimum age,^11^ others have suggested that aspects of QST can be performed with children as young as 4 years^24^ or even 2 years of age.^4^ As psychophysical tests, QST measures are sensitive to cognitive factors such as attention, concentration, and reaction time,^18^ which are factors still developing in childhood.^27,33^ These can affect the quality of QST results by increasing data variability.^12^ Although cooperation can be enhanced in a quiet calming environment and engaging through play or distraction, further research on the feasibility of performing QST with young children should be conducted.

Findings suggest that QST in pediatric populations has been limited by the number and type of sensory modalities used. The vast majority of studies in this review used only one QST modality, the most common of which was pressure. Indeed, one of the benefits of QST over other experimental pain methods (ie, the cold pressor task) is its ability to assess the functioning of distinct peripheral fibers.^5^ The narrow focus on pressure as a QST modality overlooks key information on somatosensory functioning that could otherwise be evaluated with other common modalities, such as heat, cold, vibration, or punctate stimuli. More research is needed using comprehensive QST protocols to assess the full spectrum of somatosensory functioning in pediatric populations. Regarding study paradigms, most studies were focused on characterizing the sensory profiles of the population of interest. Findings from this review suggest that there has been limited work using more advanced, dynamic QST paradigms (eg, CPM), or integrating the use of QST with other paradigms (eg, fMRI, assessing intervention efficacy) in pediatric research.

A striking finding in this review was the substantial variation in the QST methods and procedures across studies. For instance, only 14% of studies included in this review reported using a standardized QST protocol, and less than one quarter of studies reported on whether participants were instructed to watch or not during testing, or whether children's parents were present during the study testing. This is consistent with results from a recent systematic review on the use of QST in pediatric chronic pain. Schoth et al.^35^ identified significant heterogeneity in study methods and lack of information on pertinent study procedures across studies, suggesting that poor reporting is a broader issue across pediatric QST studies. Because QST relies on participants' perception, results are inherently sensitive to contextual factors, such as instructions, environment, and individuals present.^6^ By example, Hohmeister et al.^25^ examined the influence of maternal presence on children's QST responses and found that maternal presence during QST was associated with increased heat pain thresholds. Findings from this review suggest that there are ongoing challenges regarding protocol variability and reporting standards, which may be biasing results and is limiting the utility of results and comparability across settings. The field would benefit from collaborative efforts to generate sensory datasets and share data registries in the pediatric field, as well as consensus on core reporting guidelines for QST studies to enhance transparency and reproducibility.

The success of any research protocol is dependent on whether it is perceived as acceptable to participants and feasible for teams to implement. Understanding the acceptability and feasibility of experimental pain methods in pediatric research, such as QST, is particularly pertinent given the possible risks and lack of direct benefits to children taking part.^9^ The current review identified that approximately half of existing studies using QST in children reported on at least one acceptability or feasibility outcome. Reassuringly, only a handful of studies reported some type of adverse event. These were uncommon and mild in nature (eg, anxiety, fear of tissue contusion). This information may help guide research ethics boards' decisions on establishing protocol risk. The most common acceptability or feasibility outcomes reported were procedure-related (eg, completion rate, protocol duration, child's understanding/cooperation). Although the risks of QST remain low,^9^ the fear associated with certain tasks (ie, PinPrick) and the impact that this may have on protocol completion rates must be taken into consideration. Only 5 studies reported on children's satisfaction with the study procedures. The rise of patient-oriented research has highlighted the vital importance of hearing directly from patients and families regarding their medical research experiences.^15^ Research suggests that when patient perspectives on research are actively sought and integrated, findings are higher quality, more relevant, and are more likely to affect practice and policy.^14,19^ The QST field would benefit from targeted efforts to solicit feedback from patients and families on testing experiences.

Findings from this review suggest that the pediatric QST literature has been limited by significant methodological variability and lack of detailed reporting. This review points to several key recommendations for future research to advance the field of pediatric QST. First, researchers are encouraged to follow a standardized protocol that has been previously trialed or used in a population similar to the one being investigated to optimize consistency and reproducibility. In the same way, it is important that studies comment on the training and experience of those administering QST protocols and should consider and report the psychometric properties of the outcome measures reported. Overall, the field of pediatric QST would benefit from collaborative efforts to generate sensory datasets using standard protocols and share data registries in the pediatric field, as well as consensus on core reporting guidelines for QST studies to enhance transparency and reproducibility.

This review had several strengths, including the use of rigorous scoping review methodology^3,28^ and broad overview of the use of QST with children across pediatric populations. That said, there are some limitations that must be acknowledged. This review defined QST as a method requiring children to self-report their sensory experiences in response to standardized stimuli. This definition therefore excluded studies examining behavioral responses (eg, withdraw reflexes) in pediatric populations unable to provide self-report (eg, children who are nonverbal, infants). For instance, there is an important emerging body of research using adapted QST protocols to assess somatosensory functioning in children with developmental or motor impairments that preclude self-report.^7,16^ These studies were excluded from the current review, however warrant further attention. This review had a broad focus, which resulted in a high volume of titles, abstracts, full texts, and final included articles. In line with the objectives of scoping reviews,^32^ results were summarized narratively with a general overview of study characteristics, as opposed to detailed reporting of study results. Future systematic reviews with narrower focus could synthesize the literature on QST study results (eg, sensory outcomes, sample size) across various pediatric populations and assess the quality of individual studies. Finally, for the search strategy, an English-language filter was used, which may have affected the retrieval of evidence in other languages, and publication date filters were used during the 2 search updates, which do not capture materials added to the database retrospectively and may have affected the discovery of relevant evidence.

In sum, QST in pediatric populations is an emerging and rapidly growing area of pain research. Studies to date have focused primarily on school-age children and adolescents and have been limited by the number and type of sensory modalities evaluated. Future work is needed using comprehensive, standardized QST protocols to harness the full potential that this experimental method can offer to our understanding of pediatric pain.

## Disclosures

The authors declare that the research was conducted in the absence of any commercial or financial relationships that could be constructed as a potential conflict of interest.

## Supplemental digital content

Supplemental digital content associated with this article can be found online at http://links.lww.com/PR9/A227 and http://links.lww.com/PR9/A226.
